# Localization of Hydrogen Peroxide in Dormant Buds of Resistant and Susceptible Chestnut Cultivars: Changes During Gall Developmental Stages Induced by the Asian Chestnut Gall Wasp (*Dryocosmus kuriphilus*) †

**DOI:** 10.3390/plants14142089

**Published:** 2025-07-08

**Authors:** Başak Müftüoğlu, Cevriye Mert

**Affiliations:** Department of Horticulture, Faculty of Agriculture, University of Bursa Uludag, Görükle Campus, Nilüfer 16059, Bursa, Türkiye; cevmert@uludag.edu.tr

**Keywords:** chestnut gall wasp, DAB staining, hydrogen peroxide, plant defense mechanisms, dormant buds

## Abstract

Asian chestnut gall wasp (ACGW) (*Dryocosmus kuriphilus* Yasumatsu), native to China, is an invasive pest that causes significant economic losses in *Castanea* species. While some cultivars show full resistance by inhibiting insect development in buds, the underlying defense mechanisms remain unclear. In this study, the accumulation and distribution of hydrogen peroxide (H_2_O_2_) were investigated in dormant buds of chestnut cultivars that are resistant and susceptible to *D. kuriphilus* by using the 3,3′-diaminobenzidine (DAB) staining method. Buds were examined under a stereomicroscope during key stages of pest development, including oviposition, transition from egg to larva, gall induction, and gall development. Baseline levels of H_2_O_2_ were detected in all buds; however, these levels varied among cultivars, with resistant cultivars exhibiting lower basal levels. The degree of H_2_O_2_ accumulation was found to vary depending on plant–insect interaction, physiological processes, and cultivar-specific traits. Histochemical staining revealed that brown spots indicative of H_2_O_2_ accumulation were concentrated in the vascular bundles of leaf primordia and in the apical regions. In resistant hybrid cultivars, the defense response was activated at an earlier stage, while in resistant *Castanea sativa* Mill. cultivars, the response was delayed but more robust. Although consistently high levels of H_2_O_2_ were observed throughout the pest interaction in susceptible cultivars, gall development was not inhibited. During the onset of physiological bud break, increased H_2_O_2_ accumulation was observed across all cultivars. This increase was associated with endodormancy in susceptible cultivars and with both defense mechanisms and endodormancy processes in resistant cultivars. These findings highlight the significant role of H_2_O_2_ in plant defense responses, while also supporting its function as a multifunctional signaling molecule involved in gall development and the regulation of physiological processes.

## 1. Introduction

Chestnut (*Castanea* spp.) is a distinctive nut crop belonging to the family *Fagaceae* within the order Fagales, closely related to the oak (*Quercus* spp.) and beech (*Fagus* spp.) genera. Its natural distribution is limited to the Northern Hemisphere, where it is widely cultivated across various regions of Asia, Europe, and North America. In recent years, however, chestnut production has expanded beyond the Northern Hemisphere, with orchards being established in Southern Hemisphere countries such as Chile, Argentina, Australia, and New Zealand [1,2]. This expansion demonstrates the significant role of chestnuts in global agriculture and the increasing area devoted to their cultivation. The most important species include *Castanea sativa* (Mill.) in Europe, *Castanea dentata* (Borkh) in America, *Castanea crenata* (Sieb et Zucc.) in Japan, and *Castanea mollissima* (Blume) in China and Korea [3].

Chestnut is an important fruit species globally due to its nutritional value and economic potential. However, diseases and pests cause substantial yield losses and tree mortality. Major diseases include *Cryphonectria parasitica* and *Phytophthora cinnamomi*, while the most significant pest is the Asian chestnut gall wasp (ACGW), *Dryocosmus kuriphilus* Yasumatsu. Originating from China, this pest causes considerable yield losses in invaded regions. *D. kuriphilus* induces gall formation on shoots and leaves, reducing leaf area and impeding the development of fruit-bearing buds [4]. There is no chemical control method available for this pest; thus, management primarily relies on biological control and the cultivation of resistant cultivars. Biological control involves using the parasitoid *Torymus sinensis*, but this method may take a long time. Another approach is developing resistant chestnut cultivars [5].

In chestnut species, cultivars and genotypes with varying levels of susceptibility to ACGW, including those exhibiting complete resistance, have been identified [5,6,7,8,9]. *D. kuriphilus* does not differentiate between resistant and susceptible cultivars during oviposition. Although cynipid eggs and larvae were detected in the buds of resistant cultivars, the larvae failed to develop. This was associated with the hypersensitive response (HR) exhibited by plants [5,10,11,12]. The hypersensitive response is a genetic process resulting in cell death at the infection site and typically involves the generation of reactive oxygen species. Besides pathogens, HR was also observed in response to certain insect infestations [5,10,11,13,14].

Reactive oxygen species (ROS) accumulate in plants under various biotic (pathogen attacks) and abiotic (high light intensity, drought, temperature extremes, salinity, and heavy metals) stress conditions [15,16,17]. While excessive ROS can lead to oxidative damage in proteins, DNA, and lipids, they also function as signaling molecules regulating developmental and stress responses [15]. Major ROS found in plants include singlet oxygen (^1^O_2_), superoxide (O_2_^−^), hydrogen peroxide (H_2_O_2_) and hydroxyl radicals (OH^−^). Among these, H_2_O_2_ has a relatively more stable structure and is thus considered the most probable signaling ROS in the regulation of developmental and stress responses [18,19]. Consequently, H_2_O_2_ stands out as one of the most extensively studied ROS [20].

Hydrogen peroxide can be detected quantitatively and qualitatively. However, accurately quantifying H_2_O_2_ in plant organs is difficult due to its high metabolic activity, characterized by a half-life of only about 1 millisecond in plants [21,22,23]. Hydrogen peroxide can also be qualitatively localized at the tissue or cellular level. When compared to measuring H_2_O_2_ extracted from whole plant organs, this approach offers the advantage of localizing H_2_O_2_ within specific cellular regions of multicellular tissues or organs, thereby providing deeper insights into its cellular origin and function. Localization of H_2_O_2_ in plant organs relies on histochemical staining procedures. 3,3′-diaminobenzidine (DAB) is one of the most frequently utilized chemicals used for H_2_O_2_ localization in plants. After uptake by plant cells, DAB reacts with H_2_O_2_ in the presence of peroxidase to form a reddish-brown polymer [24]. DAB-mediated tissue printing has been utilized to localize peroxidase in plants [25].

Studies determining the role of H_2_O_2_ in plant disease and pest interactions using DAB staining typically focused on leaves [24,26,27,28,29,30], root cells [29,31], and gall tissues [32]. The only study assessing the hypersensitive reaction to *D. kuriphilus* in chestnut buds through DAB staining was carried out by Dini et al. [11]. In that study, the presence of H_2_O_2_ was examined via DAB staining at different stages of budburst in the resistant ‘Bouche de Bétizac’ hybrid and the susceptible ‘Madonna’ (*C. sativa*) cultivar. Brown coloration was observed in the buds of ‘Bouche de Bétizac’, whereas a whitish appearance was noted in ‘Madonna’.

Gall formation and its underlying mechanisms involve signaling molecules such as hydrogen peroxide and phytohormones. ROS are claimed to play a role in gall induction, development, and the formation of histochemical gradients. Controlled ROS levels stimulate biochemical changes and novel cellular developmental pathways, leading to gall formation through the accumulation of (poly)phenols and phytohormones (e.g., auxin) at gall sites. ROS might serve as initial signaling molecules triggering diverse events. While high concentrations of ROS can induce cell death, thereby causing unsuccessful gall formation, low ROS concentrations may lead to biochemical alterations that regulate plant cell responses and promote gall development [33].

This study aims to determine hydrogen peroxide synthesis, localization, and temporal variations occurring during plant-pest interactions within buds throughout the gall developmental process in chestnut cultivars susceptible and resistant to gall wasp infestation. The DAB staining method was employed to detect the presence of hydrogen peroxide in buds. Buds were examined under a stereomicroscope during critical phases of plant-pest interactions, namely, the pest’s oviposition period, egg-to-larva transformation, gall induction, and gall development, as pest development is inhibited within the buds.

## 2. Results

In this study, the localization and accumulation of H_2_O_2_ in the buds of chestnut cultivars classified as resistant (‘Bouche de Bétizac’, ‘Ertan’, ‘Tülü’), less susceptible (‘Maraval’), and susceptible (‘Marigoule’, ‘Alimolla’, ‘Sarıkestane’) to the ACGW were examined under a stereomicroscope from the egg-laying period of the pest through to bud burst. Moreover, the intensity of DAB staining, indicative of H_2_O_2_ accumulation, was assessed using a three-point scale (1 = weak staining; 3 = strong staining), and oxidative defense levels among cultivars were compared based on average scores (Table 1). Across all observation periods, brown staining indicative of H_2_O_2_ presence was detected in the buds.

### 2.1. Control Period

**Figure 1:**
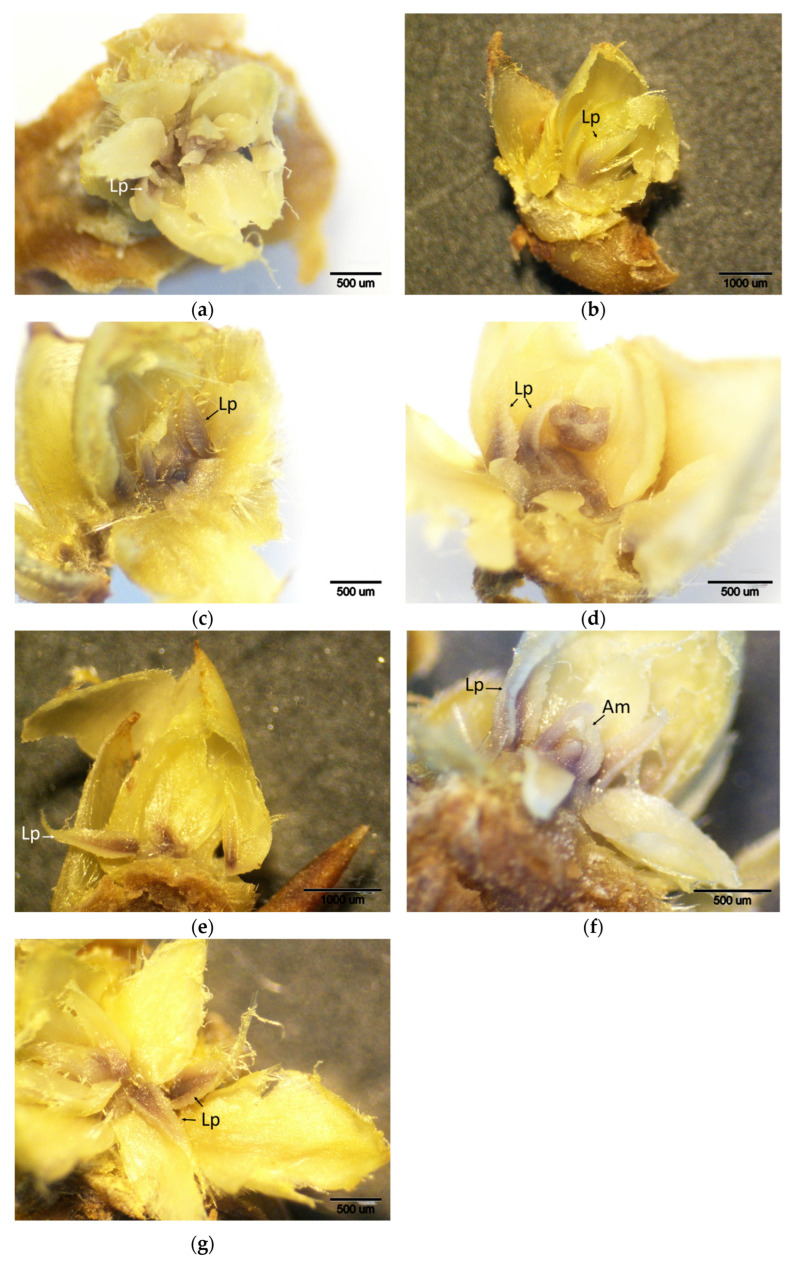
Histochemical detection (shown by the brownish reaction product) of hydrogen peroxide using DAB staining in buds of chestnut cultivars resistant and susceptible to ACGW during the control period. (**a**) Bouche de Bétizac (R); (**b**) Ertan (R); (**c**) Tülü (R); (**d**) Maraval (LS); (**e**) Marigoule (S); (**f**) Alimolla (S); (**g**) Sarıkestane (S) ((**a**,**c**,**d**,**f**,**g**): bar = 500 µm; (**b**,**e**): bar = 1000 µm). R: resistant; LS: less susceptible; S: susceptible; Am: apical meristem; Lp: leaf primordia.

During the control period (when buds were not yet infested), brown-stained regions localized in the apical meristems and leaf primordia were observed in all chestnut cultivars, indicating basal levels of H_2_O_2_ accumulation as revealed by DAB staining (Figure 1). These observations suggest a low or moderate-level oxidative response occurring during bud development, independent of pest activity. DAB scores showed significant variation among cultivars. The lowest H_2_O_2_ accumulation was observed in the resistant cultivars ‘Ertan’ (1.00), ‘Tülü’ (1.66), and ‘Bouche de Bétizac’ (1.50). In contrast, higher scores were observed in the less susceptible ‘Maraval’ (2.50) and in the susceptible cultivars ‘Marigoule’ (2.16), ‘Alimolla’ (2.16), and ‘Sarıkestane’ (2.00) (Table 1).

### 2.2. ACGW Oviposition Period (t1 and t2 Stages)

**Figure 2:**
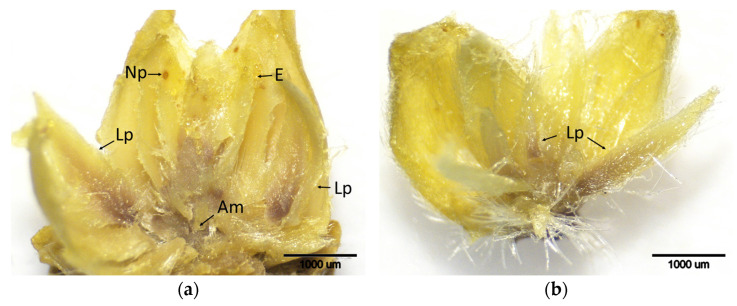

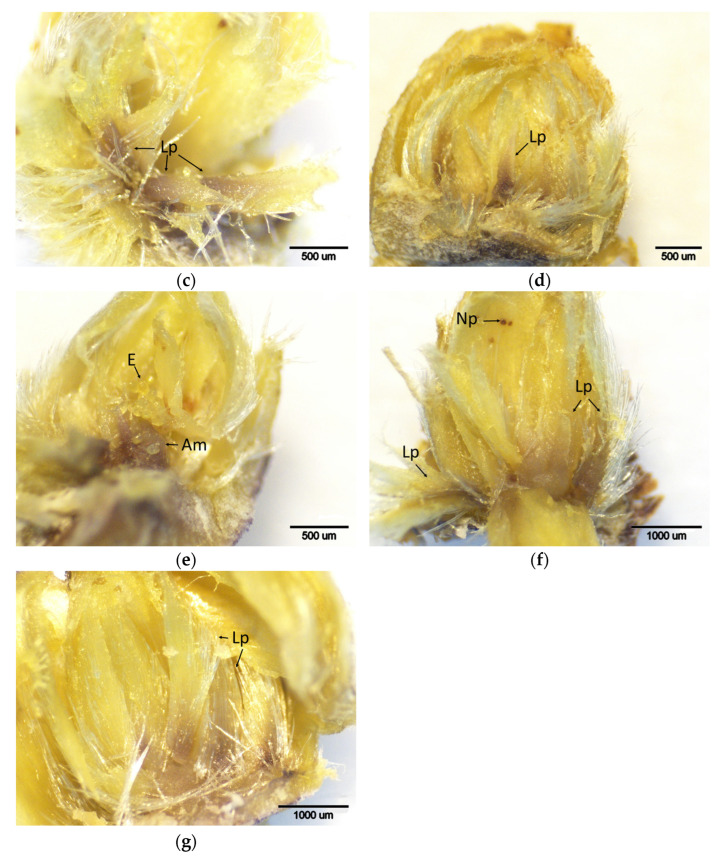
Histochemical detection (shown by the brownish reaction product) of hydrogen peroxide using DAB staining in buds of chestnut cultivars resistant and susceptible to ACGW at the initial period of ACGW infestation and egg deposition in the buds (t1). (**a**) Bouche de Bétizac (R); (**b**) Ertan (R); (**c**) Tülü (R); (**d**) Maraval (LS); (**e**) Marigoule (S); (**f**) Alimolla (S); (**g**) Sarıkestane (S) ((**a**,**b**,**f**,**g**): bar = 1000 µm; (**c**,**d**,**e**): bar = 500 µm). R: resistant; LS: less susceptible; S: susceptible; Am: apical meristem; E: eggs; Lp: leaf primordia; Np: needle puncture.

**Figure 3:**
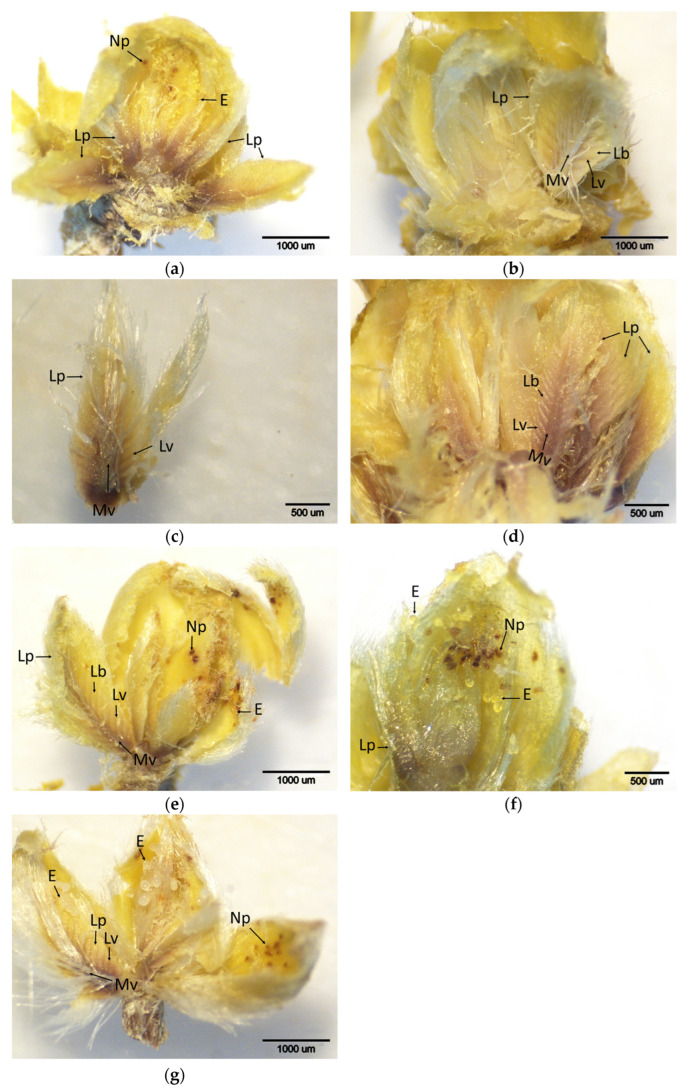
Histochemical detection (shown by the brownish reaction product) of hydrogen peroxide using DAB staining in buds of chestnut cultivars resistant and susceptible to ACGW during the period of intensive ACGW infestation (t2). (**a**) Bouche de Bétizac (R); (**b**) Ertan (R); (**c**) Tülü (R); (**d**) Maraval (LS); (**e**) Marigoule (S); (**f**) Alimolla (S); (**g**) Sarıkestane (S) ((**a**,**b**,**e**,**g**): bar = 1000 µm; (**c**,**d**,**f**): bar = 500 µm). R: resistant; LS: less susceptible; S: susceptible; E: eggs; Lb: leaf blades; Lp: leaf primordia; Lv: leaf vein; Mv: mid vein; Np: needle puncture.

During the initial ACGW infection phase (t1), when egg-laying started, and the subsequent stage (t2), when buds were heavily infested with eggs, differences in the intensity of brown staining were noted among cultivars (Figure 2 and Figure 3). At stage t2, particularly in the leaf primordia, the extent of brown-stained areas increased, indicating elevated H_2_O_2_ accumulation. This increase was prominent in all cultivars except for the resistant ‘Ertan’ (Figure 3). DAB staining scores at both stages differed significantly among cultivars (Table 1). There was a significant increase in DAB scores in ‘Bouche de Bétizac’ and ‘Maraval’ during t2. These results suggest that resistant and less susceptible cultivars may mount early oxidative defense responses capable of limiting gall formation. On the other hand, the absence of a notable increase in H_2_O_2_ accumulation in ‘Ertan’ during stage t2 may indicate that this cultivar employs an alternative defense strategy with minimal oxidative stress.

### 2.3. Gall Induction (t3) and Early Morphogenesis (t4)

**Figure 4:**
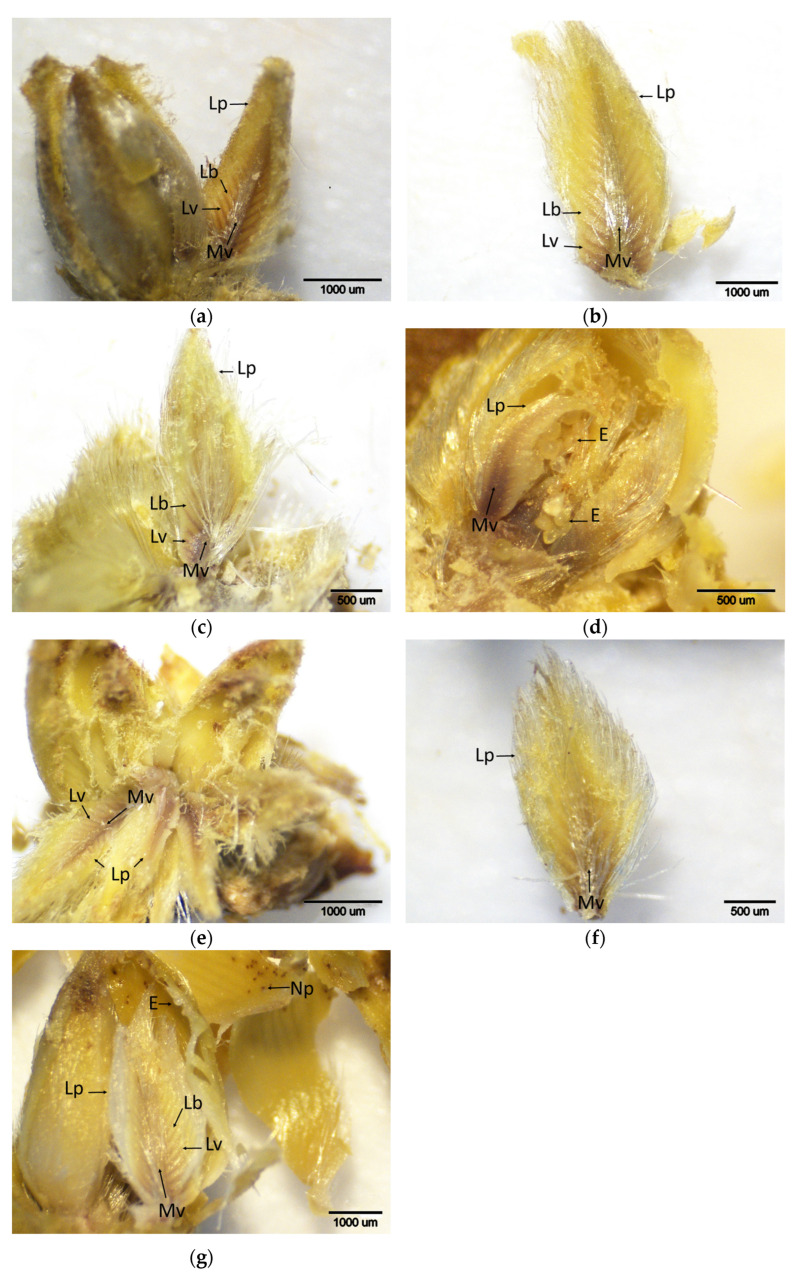
Histochemical detection (shown by the brownish reaction product) of hydrogen peroxide using DAB staining in buds of chestnut cultivars resistant and susceptible to ACGW at the developmental stage when eggs transition to larvae (t3). (**a**) Bouche de Bétizac (R); (**b**) Ertan (R); (**c**) Tülü (R); (**d**) Maraval (LS); (**e**) Marigoule (S); (**f**) Alimolla (S); (**g**) Sarıkestane (S) ((**a**,**b**,**e**,**g**): bar = 1000 µm; (**c**,**d**,**f**): bar = 500 µm). R: resistant; LS: less susceptible; S: susceptible; E: eggs; Lb: leaf blades; Lp: leaf primordia; Lv: leaf vein; Mv: midvein; Np: needle puncture.

**Figure 5:**
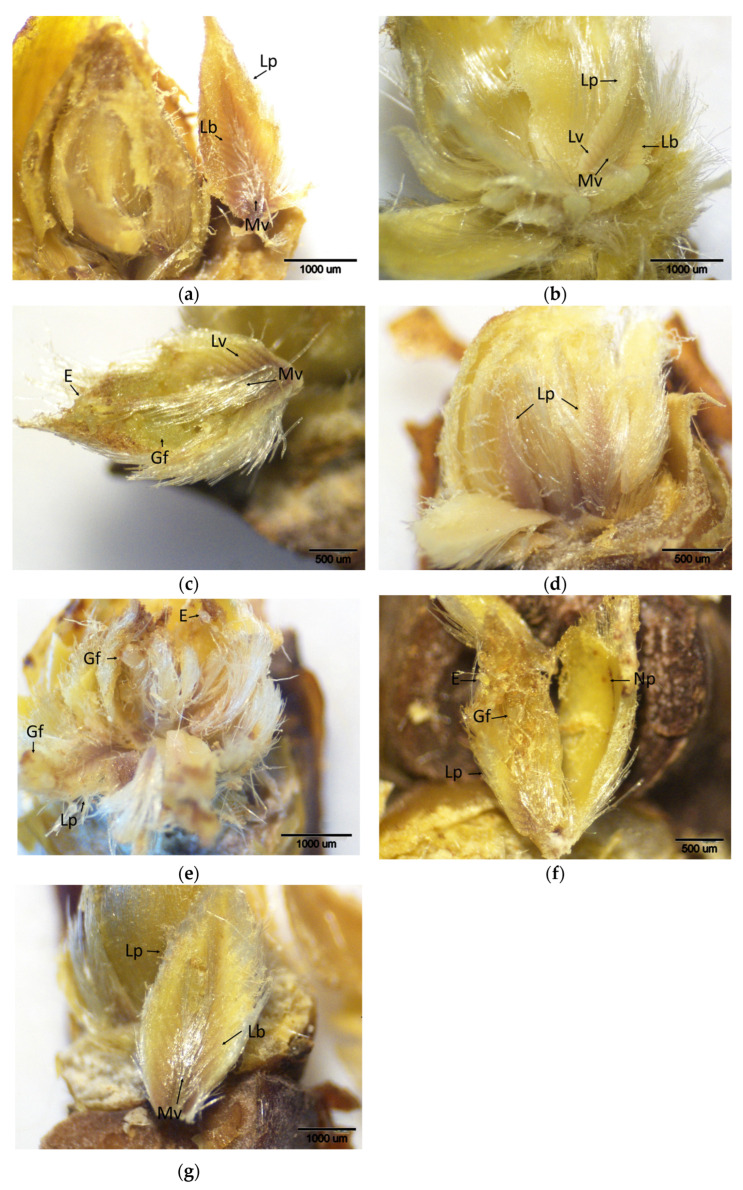
Histochemical detection (shown by the brownish reaction product) of hydrogen peroxide using DAB staining in buds of chestnut cultivars resistant and susceptible to ACGW at the onset of gall tissue formation (t4). (**a**) Bouche de Bétizac (R); (**b**) Ertan (R); (**c**) Tülü (R); (**d**) Maraval (LS); (**e**) Marigoule (S); (**f**) Alimolla (S); (**g**) Sarıkestane (S) ((**a**,**b**,**e**,**g**): bar = 1000 µm; (**c**,**d**,**f**): bar = 500 µm). R: resistant; LS: less susceptible; S: susceptible; E: eggs; Gf: gall formation; Lb: leaf blades; Lp: leaf primordia; Lv: leaf vein; Mv: mid vein; Np: needle puncture.

Significant differences in DAB staining intensity among cultivars were observed during the gall induction stage (t3) and early morphogenesis (t4). In particular, intense brown staining along the main and lateral veins of the leaf primordia was prominent in the hybrid cultivars ‘Bouche de Bétizac’, ‘Maraval’, and ‘Marigoule’ (Figure 4 and Figure 5). Mean DAB scores for these stages revealed statistically significant differences between cultivars (Table 1). Hybrid cultivars exhibited the highest average score (2.5), whereas cultivars belonging to *C. sativa*, including ‘Alimolla’ (2.33), ‘Sarıkestane’ (2.16), and ‘Tülü’ (1.66), yielded lower scores. The lowest score was again observed in the *C. sativa* cultivar ‘Ertan’ (1.16). These results indicate cultivar-dependent variability in H_2_O_2_ accumulation and the associated oxidative defense responses.

### 2.4. Advanced Gall Development Stage

**Figure 6:**
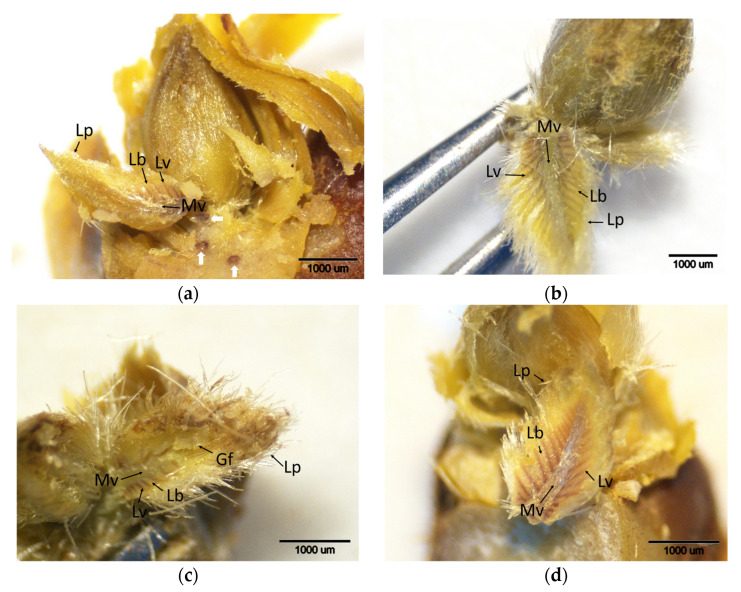

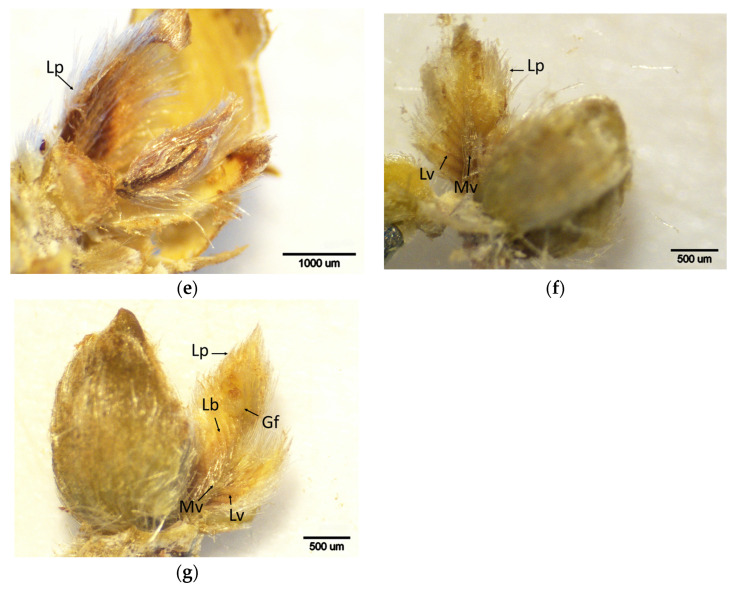
Histochemical detection (shown by the brownish reaction product) of hydrogen peroxide using DAB staining in buds of chestnut cultivars resistant and susceptible to ACGW during September. (**a**) Bouche de Bétizac (R); (**b**) Ertan (R); (**c**) Tülü (R); (**f**) Alimolla (S); (**g**) Sarıkestane (S)—the presence of H_2_O_2_ can be observed in the vascular bundles of the regions indicated by the white arrows, such areas representing the points where leaf primordia have detached; (**d**) Maraval (LS); (**e**) Marigoule (S) ((**a**,**b**,**c**,**d**,**e**): bar = 1000 µm; (**f**,**g**): bar = 500 µm). R: resistant; LS: less susceptible; S: susceptible; Gf: gall formation; Lb: leaf blades; Lp: leaf primordia; Lv: leaf vein; Mv: mid vein.

**Figure 7:**
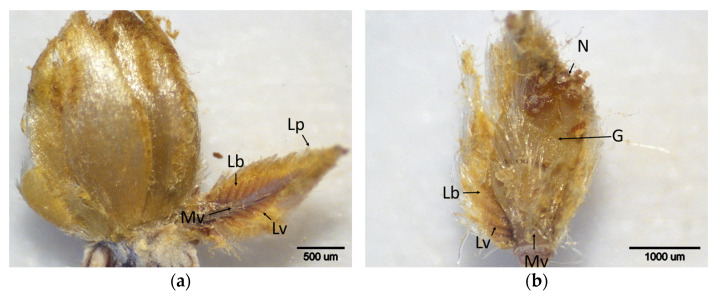

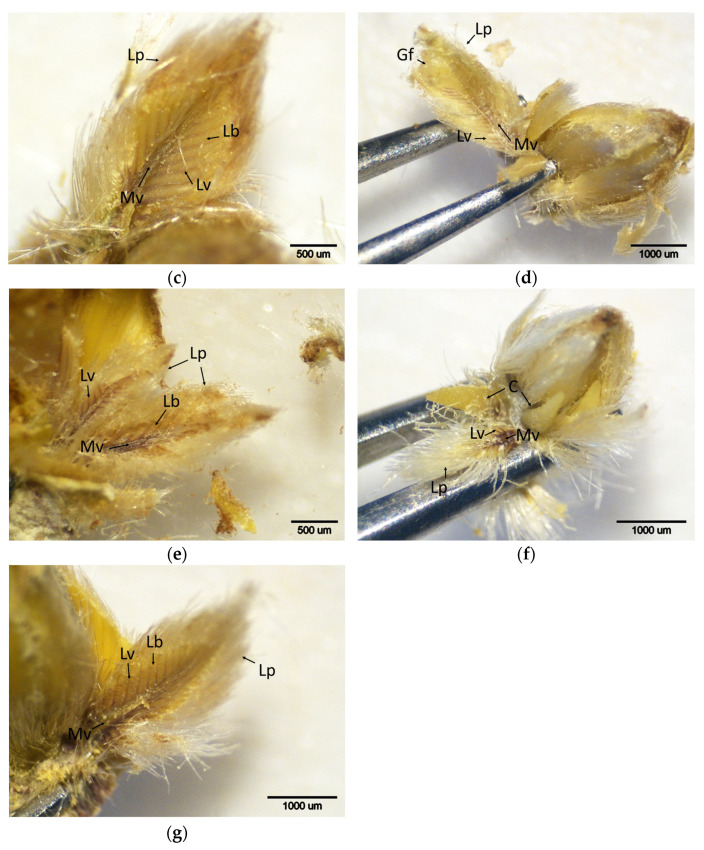
Histochemical detection (shown by the brownish reaction product) of hydrogen peroxide using DAB staining in buds of chestnut cultivars resistant and susceptible to ACGW during October. (**a**) Bouche de Bétizac (R); (**b**) Ertan (R); (**c**) Tülü (R); (**d**) Maraval (LS); (**e**) Marigoule (S); (**f**) Alimolla (S); (**g**) Sarıkestane (S) ((**a**,**c**,**e**): bar = 500 µm; (**b**,**d**,**f**,**g**): bar = 1000 µm). R: resistant; LS: less susceptible; S: susceptible; C: catkin; G: gall; Gf: gall formation; Lb: leaf blades; Lp: leaf primordia; Lv: leaf vein; Mv: midvein; N: necrosis.

**Figure 8:**
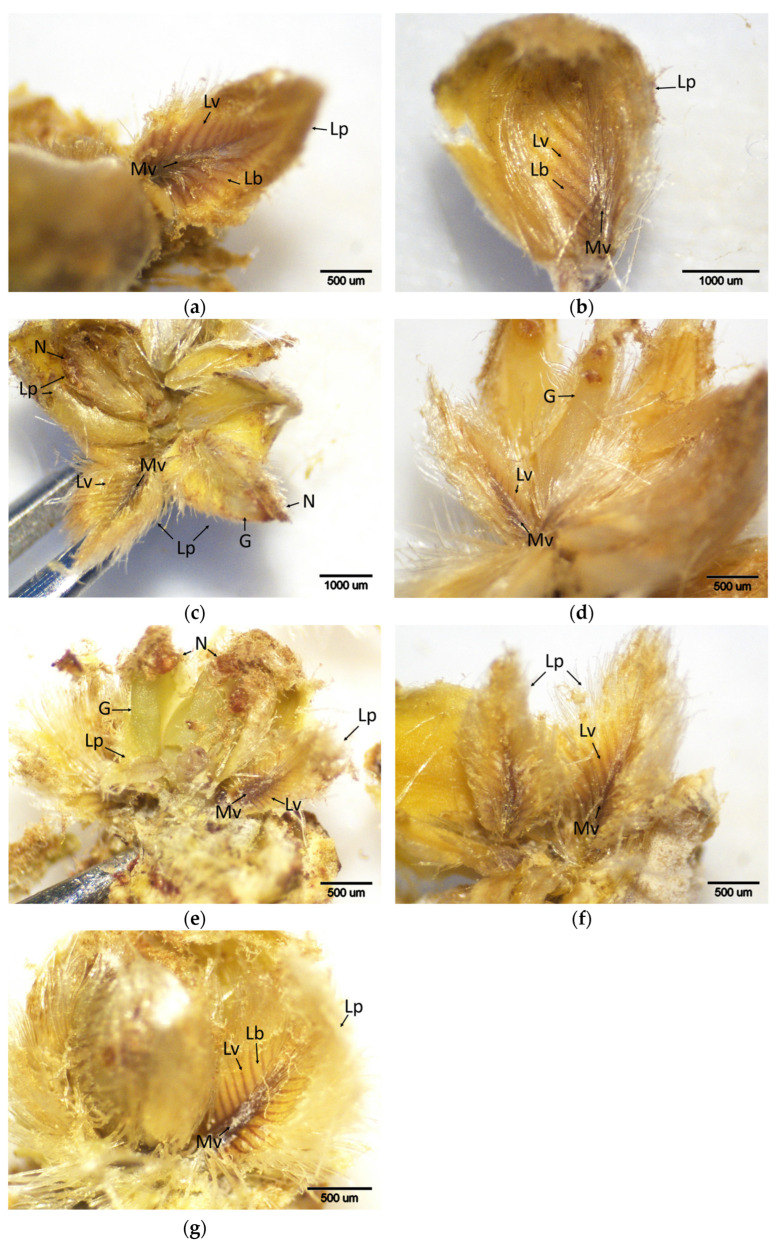
Histochemical detection (shown by the brownish reaction product) of hydrogen peroxide using DAB staining in buds of chestnut cultivars resistant and susceptible to ACGW during December. (**a**) Bouche de Bétizac (R); (**b**) Ertan (R); (**c**) Tülü (R); (**d**) Maraval (LS); (**e**) Marigoule (S); (**f**) Alimolla (S); (**g**) Sarıkestane (S) ((**a**,**d**,**e**,**f**,**g**): bar = 500 µm; (**b**,**c**): bar = 1000 µm). R: resistant; LS: less susceptible; S: susceptible; G: gall; Lb: leaf blades; Lp: leaf primordia; Lv: leaf vein; Mv: mid vein; N: necrosis.

In September, brown staining in the majority of leaf primordia was observed across all cultivars (Figure 6). Notably, the staining intensity increased in the resistant cultivar ‘Ertan’. Necrotic lesions were also identified in galls formed on the leaf primordia of all cultivars. During October and December, the severity of staining remained similar to that observed in September, with necrotic lesions (indicative of cell death) persisting in the galls of resistant cultivars (Figure 7 and Figure 8). In contrast, gall development continued in the susceptible cultivars.

In September, high DAB scores were recorded in both resistant and susceptible cultivars, ranging from 2.00 to 2.66, and these elevated levels persisted through October and December (Table 1). However, statistical analysis indicated that differences among cultivars were significant only in September and not in the following months. Between stage t4 and September, a remarkable increase in DAB scores was observed in ‘Tülü’ and, notably, in the resistant cultivar ‘Ertan’, with increases of 50.60% and 72.41%, respectively. This finding suggests that the resistant *C. sativa* cultivars ‘Tülü’ and ‘Ertan’, which exhibited a limited oxidative response during stages t2 and t3, activated their defense mechanisms more prominently during later stages of gall development, particularly in September. In contrast, the increase observed in other cultivars during this period was less pronounced.

**Figure 9:**
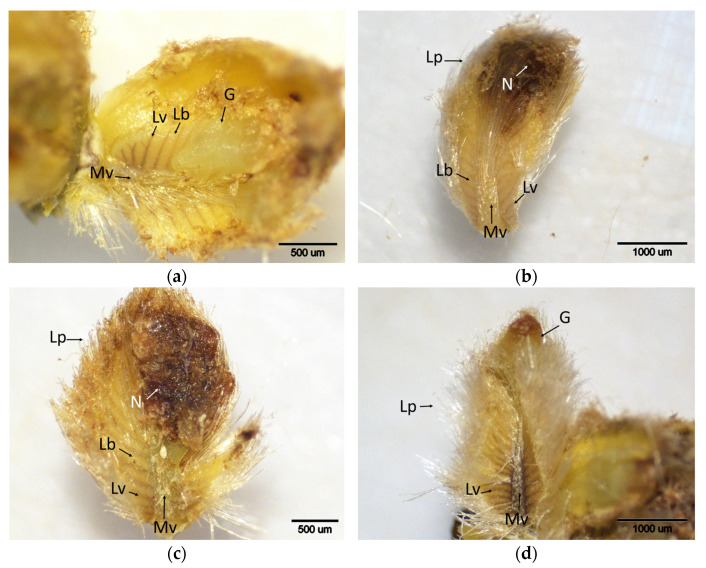

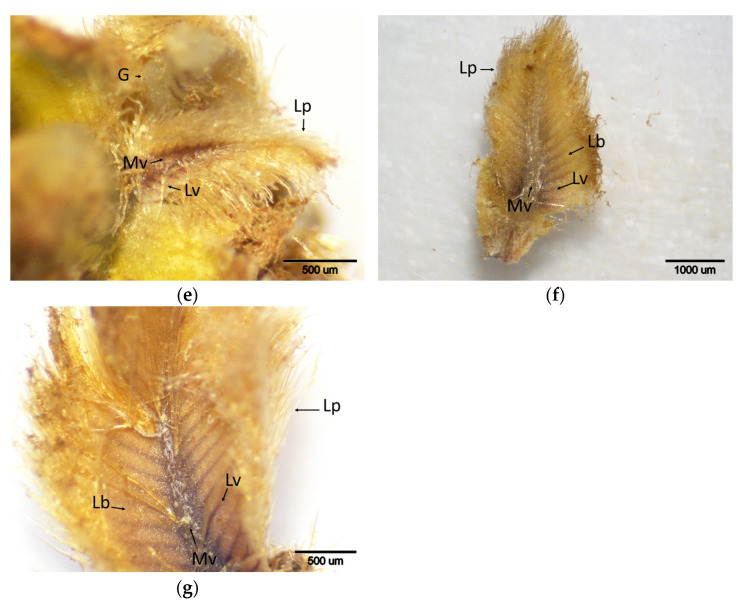
Histochemical detection (shown by the brownish reaction product) of hydrogen peroxide using DAB staining in buds of chestnut cultivars resistant and susceptible to ACGW during January. (**a**) Bouche de Bétizac (R); (**b**) Ertan (R); (**c**) Tülü (R); (**d**) Maraval (LS); (**e**) Marigoule (S); (**f**) Alimolla (S); (**g**) Sarıkestane (S) ((**a**,**c**,**e**,**g**): bar = 1000 µm; (**b**,**d**,**f**): bar = 500 µm). R: resistant; LS: less susceptible; S: susceptible; G: gall; Lb: leaf blades; Lp: leaf primordia; Lv: leaf vein; Mv: mid vein; N: necrosis.

In January, a reduction in DAB staining intensity was observed in the buds of resistant cultivars (Figure 9). In the buds of the resistant cultivars ‘Ertan’, ‘Tülü’, and ‘Bouche de Bétizac’, faint brown spots were detected in the leaf primordia. In contrast, the less susceptible ‘Maraval’ and the susceptible cultivars ‘Sarıkestane’, ‘Alimolla’, and ‘Marigoule’ yielded more pronounced brown staining, particularly along the main veins of the leaf primordia. During the same period, necrosis and darkening associated with cell death were observed in galls developing within the leaf primordia of resistant cultivars. This phenomenon was especially evident in the *C. sativa* cultivars ‘Tülü’ and ‘Ertan’, suggesting that the defense response was activated in a manner that restricted gall tissue development. Conversely, in the susceptible cultivars, both continued gall development and intense DAB staining were noted in the leaf primordia. This suggests that H_2_O_2_ accumulation may be associated with active gall formation and pest development.

**Figure 10:**
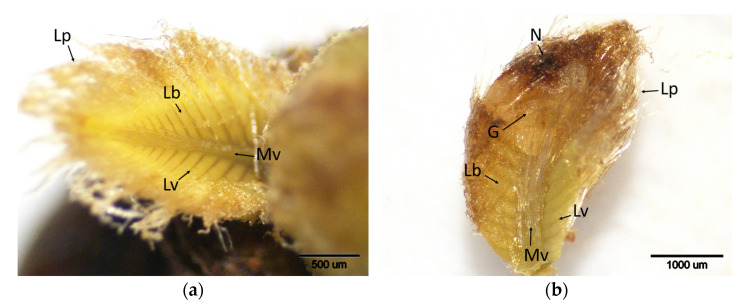

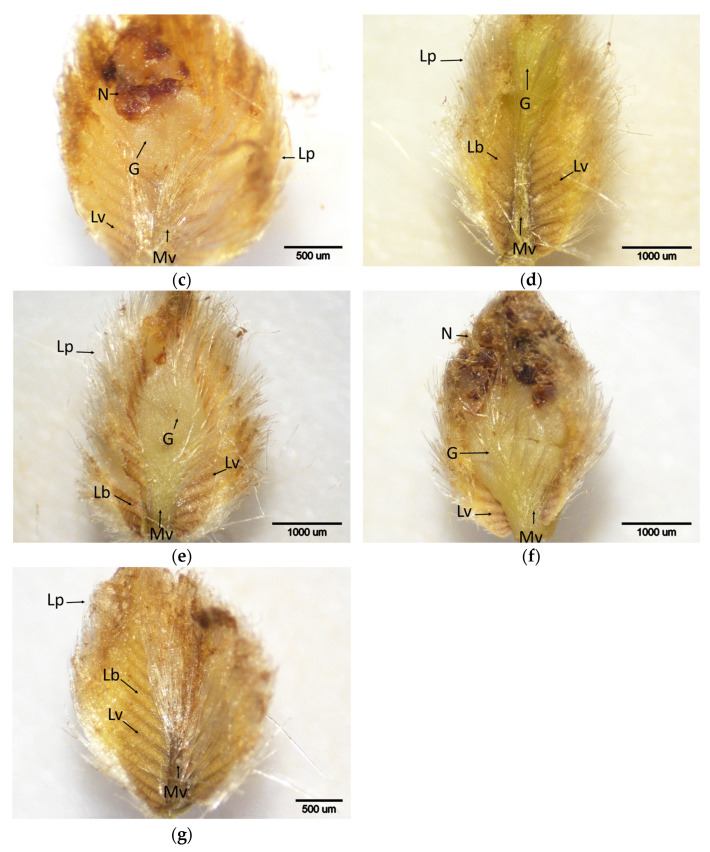
Histochemical detection (shown by the brownish reaction product) of hydrogen peroxide using DAB staining in buds of chestnut cultivars resistant and susceptible to ACGW during February. (**a**) Bouche de Bétizac (R); (**b**) Ertan (R); (**c**) Tülü (R); (**d**) Maraval (LS); (**e**) Marigoule (S); (**f**) Alimolla (S); (**g**) Sarıkestane (S) ((**a**,**c**,**g**): bar = 500 µm; (**b**,**d**,**e**,**f**): bar = 1000 µm). R: resistant; LS: less susceptible; S: susceptible; G: gall; Lb: leaf blades; Lp: leaf primordia; Lv: leaf vein; Mv: mid vein; N: necrosis.

In February, DAB staining intensity remained similar to that observed in January (Figure 10). A statistical analysis of the DAB scores for January and February revealed significant differences among cultivars (Table 1). During this period, low DAB scores and mild staining were observed in resistant cultivars, while high scores and intense staining were detected in susceptible ones. These results indicate that resistant cultivars respond to gall development with oxidative defense mechanisms that lead to cell death and gall necrosis, thereby limiting pest spread. In contrast, susceptible cultivars exhibited increased H_2_O_2_ accumulation in actively developing gall tissues.

**Figure 11:**
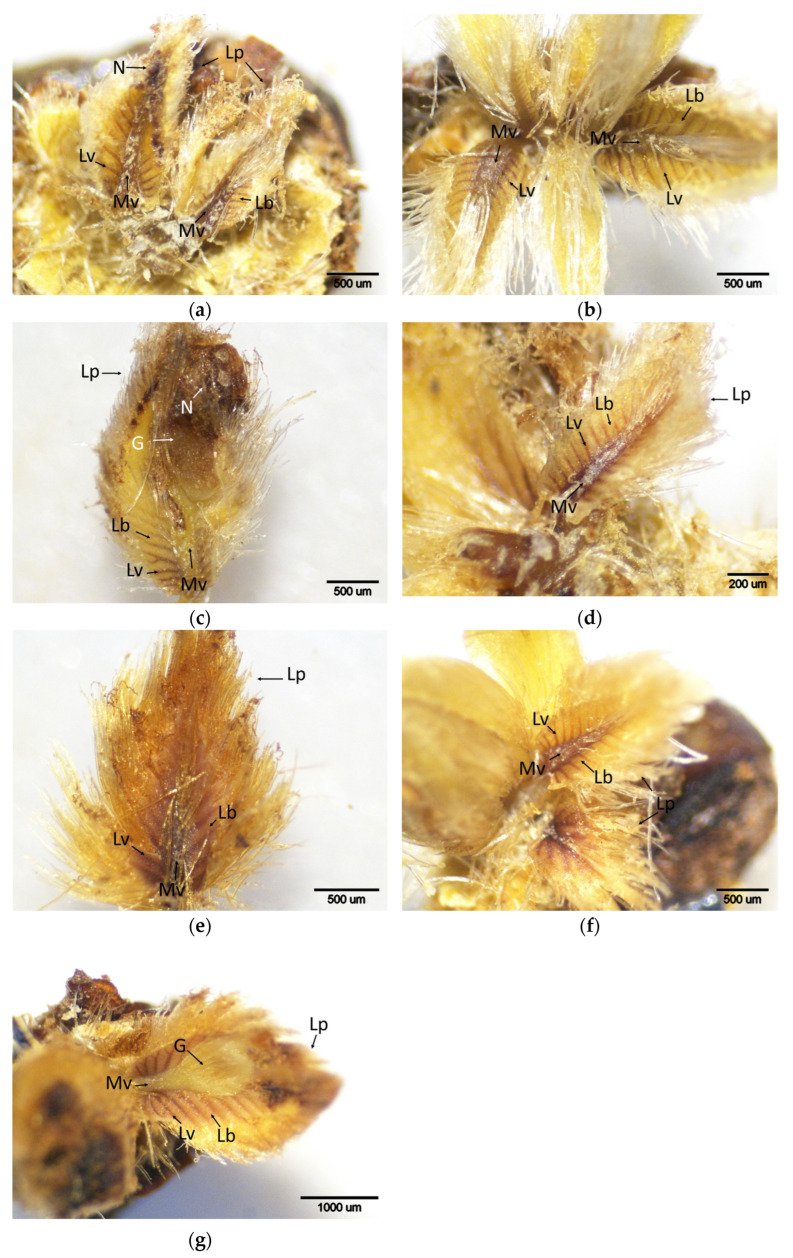
Histochemical detection (shown by the brownish reaction product) of hydrogen peroxide using DAB staining in buds of chestnut cultivars resistant and susceptible to ACGW during March. (**a**) Bouche de Bétizac (R); (**b**) Ertan (R); (**c**) Tülü (R); (**d**) Maraval (LS); (**e**) Marigoule (S); (**f**) Alimolla (S); (**g**) Sarıkestane (S) ((**a**,**b**,**c**,**e**,**f**): bar = 500 µm; (**d**): bar = 200 µm; g: bar = 1000 µm). R: resistant; LS: less susceptible; S: susceptible; G: gall; Lb: leaf blades; Lp: leaf primordia; Lv: leaf vein; Mv: mid vein; N: necrosis.

In March, the phenological stage of bud swelling was observed at different dates across all cultivars. During this period, physiological bud awakening led to increased metabolic activity, initiating cell division and growth processes. At this transitional stage, prominent brown staining indicating hydrogen peroxide accumulation was detected in the leaf primordia of buds from all cultivars (Figure 11). The staining was widespread throughout the leaf primordia, with particularly intense brown coloration along the main and lateral veins. This increase in March was also reflected in DAB scores, with the highest rates of increase recorded in the resistant cultivars ‘Ertan’ (126%), ‘Tülü’ (129%), and ‘Bouche de Bétizac’ (100%) (Table 1). These findings indicate that oxidative defense mechanisms in these cultivars were activated not only in response to pest presence but also in synchrony with the growth phase. High DAB scores suggest that the H_2_O_2_-mediated defense response was effectively functioning within gall tissues.

**Figure 12:**
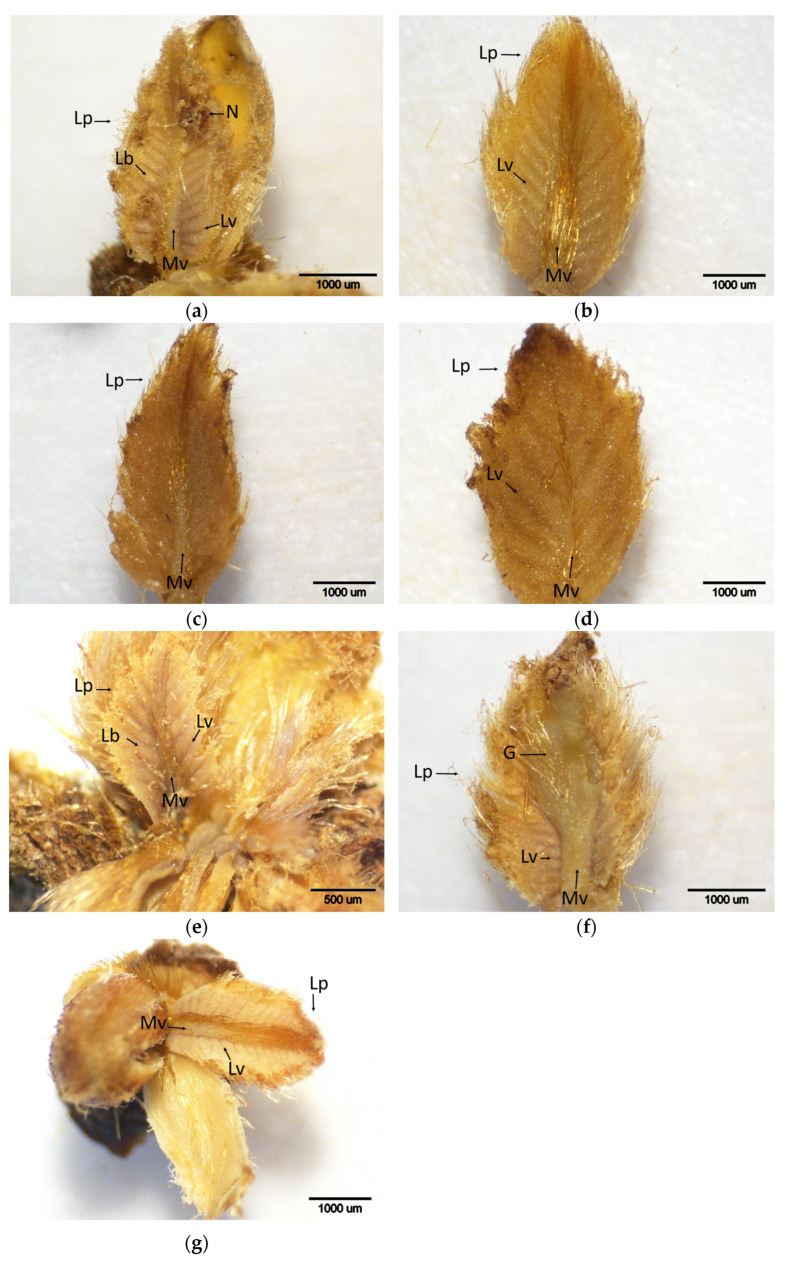
Histochemical detection (shown by the brownish reaction product) of hydrogen peroxide using DAB staining in buds of chestnut cultivars resistant and susceptible to ACGW during April. (**a**) Bouche de Bétizac (R); (**b**) Ertan (R); (**c**) Tülü (R); (**d**) Maraval (LS); (**e**) Marigoule (S); (**f**) Alimolla (S); (**g**) Sarıkestane (S) ((**a**,**b**,**c**,**d**,**f**,**g**): bar = 1000 µm; (**e**): bar = 500 µm). R: resistant; LS: less susceptible; S: susceptible; G: gall; Lb: leaf blades; Lp: leaf primordia; Lv: leaf vein; Mv: mid vein.

In the following days (approximately 9–20 days later), the phenological stage of bud burst was recorded. During this stage, DAB staining in the sampled buds decreased remarkably, with only faint coloration observed (Table 1, Figure 12). These observations indicate that H_2_O_2_ accumulation plays a significant role, particularly during the bud break phase (release from endodormancy), but that the oxidative response is suppressed during the bud burst stage.

## 3. Discussion

Reactive oxygen species are fundamental components of the plant defense system, and their interactions play a critical role in determining the effectiveness of defense responses. Among these, H_2_O_2_ serves as a central signaling molecule, interacting with other ROS such as superoxide anion (O_2_^−^), hydroxyl radical (OH•), and singlet oxygen (^1^O_2_). These molecules can interconvert, thereby regulating intracellular redox balance and triggering the activation of defense-related genes. In particular, the enzymatic conversion of superoxide into H_2_O_2_ by superoxide dismutase (SOD) underscores the pivotal role of these interactions in the defense process [34]. Changes in hydrogen peroxide levels are recognized as a significant indicator for monitoring fluctuations in cellular metabolism and stress responses [35]. In this study, hydrogen peroxide synthesis, localization, and temporal changes in the buds of resistant and susceptible cultivars of both hybrid (*C. sativa* × *C. crenata*) and pure *C. sativa* species against *D. kuriphilus* were investigated in detail using histochemical staining methods under microscopy, yielding substantial data. Furthermore, the visualization of H_2_O_2_ accumulation in tissues using the DAB staining method highlights the central role of this molecule in plant defense and provides a semi-quantitative approach to understanding ROS signaling.

Hydrogen peroxide presence was consistently detected in the buds of all examined chestnut cultivars. The observation of H_2_O_2_ accumulation even in the absence of pest interaction during the control period suggests that a low or moderate-level oxidative response physiologically occurs as part of the developmental processes. The differences in DAB staining intensity among cultivars during this period indicate that their ability to regulate oxidative balance may be associated with their respective levels of resistance. In particular, the lower accumulation of H_2_O_2_ in resistant cultivars implies a more effective control of oxidative stress, potentially due to a more robust antioxidant defense system.

External staining was not observed in any cultivar during the examination period; staining was instead localized in the apical meristem and leaf primordia adjacent to the apical meristem. Brown staining was observed to be especially concentrated in vascular bundles, particularly within the main and lateral veins of the leaf primordia. This regional accumulation indicates that H_2_O_2_ production and translocation are not random but rather confined to specific tissues. Leaf veins not only function in the transport of water and nutrients but may also act as conduits for defense-related signals (e.g., salicylic acid (SA), jasmonic acid (JA) and ethylene (ET)) and serve as centers for initiating localized defense responses [36].

Recent studies suggest that systemic defense responses depend on phloem-associated signaling and that ROS can be transported by the phloem [32,37]. Microscopic studies employing specific fluorescent dyes demonstrated ROS accumulation in the vascular bundles of various plant species under different stress conditions. Fluorescent staining is often more pronounced in vascular bundles when compared to other tissues [38,39]. Specifically, research by [32] histochemically detected H_2_O_2_ reactions in galls induced by *Espinosa nothofagi* in *Nothofagus obliqua* buds, observing intense H_2_O_2_ accumulation in vascular bundles, cell walls, and the apoplast of nutritive tissue. Similarly, the present study confirmed through histochemical staining that H_2_O_2_ is localized and transported within vascular bundles. These findings demonstrate the critical role of H_2_O_2_ in plant defense responses and systemic signal transduction, highlighting its accumulation and movement primarily within vascular tissues.

Differences were observed between cultivars regarding hydrogen peroxide intensity and distribution. Hybrid cultivars exhibited more pronounced staining in the buds. Additionally, the intensity of brown staining indicative of H_2_O_2_ accumulation varied significantly among resistant cultivars. Particularly, intense staining was noted in the cultivar ‘Bouche de Bétizac’, whereas staining was notably weaker in ‘Tülü’ and particularly slight in ‘Ertan’. These findings indicate that hydrogen peroxide accumulation and defense mechanisms operate at varying levels across different cultivars.

During the period of intense bud infestation by ovipositing females(t2), the increase in DAB staining scores in the resistant cultivar ‘Bouche de Bétizac’ and the less susceptible ‘Maraval’ suggests that these cultivars can restrict gall formation by initiating early oxidative defense responses. In contrast, the remarkable increase in DAB staining observed in September in the resistant *C. sativa* cultivars ‘Ertan’ and ‘Tülü’, along with the presence of necrotic lesions, indicates a delayed but effective defense response. This response is thought to be associated with a programmed cell death mechanism activated by H_2_O_2_. In susceptible cultivars, despite high levels of H_2_O_2_ accumulation, gall development persists, indicating that the oxidative response alone is insufficient for effective defense. This highlights that H_2_O_2_ is not merely a marker of stress but also that its effect depends on timing, concentration, and the cultivar’s inherent defense capacity.

A general reduction in H_2_O_2_ levels was observed in January. During this period, low staining intensity and signs of cell death (necrosis) in gall tissues were detected in resistant cultivars. This suggests that resistant cultivars develop a more balanced and effective defense response by maintaining controlled H_2_O_2_ accumulation. On the other hand, in less susceptible and susceptible cultivars, gall development in leaf primordia continued alongside H_2_O_2_ accumulation, supporting a direct relationship between H_2_O_2_ accumulation and both gall formation and pest development.

Literature data indicate that H_2_O_2_ not only causes oxidative damage but also functions as a subcellular signaling molecule during the early stages of gall formation [40,41]. Moreover, even though antioxidant defense mechanisms (e.g., catalase (CAT), peroxidase (POD), proline, anthocyanins, phenolics) are activated in gall tissues, H_2_O_2_ accumulation remains high [42]. Another study explains that changes in the gene expression of antioxidant enzymes (SOD, POD, CAT) help regulate H_2_O_2_ levels, thereby limiting toxicity while modulating defense signaling pathways [43].

H_2_O_2_ is not merely a byproduct of oxidative stress; it is a central component of a complex signaling network involving phytohormones such as auxins, gibberellins, cytokinins, abscisic acid, JA, ET, SA, as well as nitrogen monoxide, Ca^2+^ and brassinosteroids. Through this network, mitogen activated protein kinase cascades and transcription factors such as WRKY and MYB are activated, leading to the expression of defense-related genes, the synthesis of phenolic compounds, and the initiation of programmed cell death [36]. In conclusion, the effectiveness of H_2_O_2_ in plant defense is determined not only by its quantity but also by its integration into a timely and spatially coordinated signaling network. This study demonstrates that resistant and less susceptible cultivars are able to utilize H_2_O_2_-mediated defense mechanisms in a more controlled and functional manner, whereas this system appears to be impaired in susceptible cultivars.

With the physiological onset of bud break in March (bud swelling), an increase in H_2_O_2_ accumulation was observed across all cultivars, with a more pronounced increase in those resistant to ACGW. This finding suggests that H_2_O_2_ functions not only as a component of the plant’s defense responses but also serves as a signaling molecule involved in developmental processes such as bud break. In April, following bud burst, a marked decline in H_2_O_2_ levels was recorded, supporting the idea that H_2_O_2_ plays a transient and regulatory role in the transition from endodormancy to active growth. This finding is consistent with previous reports indicating that H_2_O_2_ acts as a signal triggering bud break in other species, such as grapevine [44] and Japanese pear [45]. On the other hand, in a study carried out by [11], DAB staining conducted at different stages of bud break revealed intense brown staining (indicative of H_2_O_2_ presence) only in the resistant cultivar ‘Bouche de Bétizac’, not in the susceptible ‘Madonna’ cultivar. In contrast, the present study, which also included ‘Bouche de Bétizac’, reports staining patterns indicative of H_2_O_2_ presence in both resistant and susceptible cultivars at all stages. This discrepancy may originate from differences in developmental timing or methodological variations.

Overall, the results achieved in this study demonstrate that H_2_O_2_ fulfills a dual function, acting both as a developmental signal and as a mediator of stress responses.

## 4. Materials and Methods

### 4.1. Plant Materials and Sampling

The chestnut cultivars used in this study are located in the Chestnut Collection Orchard in the Cumalıkızık region of Bursa Province. This orchard has been heavily infested by ACGW. The susceptibility levels of cultivars with favorable organoleptic characteristics to ACGW were previously identified in an earlier study [5]. In this investigation, the ACGW-resistant cultivars ‘Bouche de Bétizac’, ‘Ertan’, and ‘Tülü’, the less susceptible cultivar ‘Maraval’, and the susceptible cultivars ‘Marigoule’, ‘Alimolla’, and ‘Sarıkestane’ were examined.

Since the development of the pest is inhibited within the buds, the localization and accumulation of H_2_O_2_ in the buds were studied from the time of egg deposition until the bud burst. Bud samples were collected during five critical periods significant for pest-plant interaction:

Control period: Period without adult flight and with no bud infestation.

t1: Period when ACGW adults laid their first eggs in the buds.

t2: Period of intensive egg infestation in buds.

t3: Period when the eggs had developed into larvae and initiation of gall induction.

t4: Period during which necrotic lesions (darkening of meristematic tissues surrounding the larva) appeared in resistant cultivars.

In addition to these periods, samples and examinations were conducted during September, October, December, January, February, March, and April. Phenological observations were made on chestnut cultivars within the experimental orchard, and when necessary, histological examinations under a stereomicroscope were carried out using the bud-opening method (removal of bud scales individually). Microscope observations allowed the determination of critical stages, including egg deposition (t1 and t2), larval development (t3), and tissue necrosis (necrotic formations, t4). Additionally, yellow sticky traps were installed in the orchards to more accurately determine the timing of stages t1, t2, and t3. These traps monitored adult emergence, peak flight periods, and the decline in adult flight activity. Shoot samples taken at sampling times were transported to the laboratory in a cold container (+4 °C). Buds were isolated from shoots on the same day, and endogenous peroxidase enzyme-based histochemical staining was performed for in situ detection of H_2_O_2_. The localization and accumulation of H_2_O_2_ in buds were then examined in detail using a stereomicroscope (SZ6045TR; Olympus Optical Co. Ltd., Tokyo, Japan).

### 4.2. Histochemical Detection of Hydrogen Peroxide Synthesis

To detect the presence of H_2_O_2_ in the buds, the DAB staining method was applied [11,34]. A minimum of six buds per cultivar were used for each sampling period. Buds, along with a small portion of woody tissue, were placed in 2 mL Eppendorf tubes filled with DAB solution. The tubes were then incubated under vacuum in darkness within a desiccator for 14–16 h. After incubation, samples were placed in a 99% ethanol bath at 95 °C for 30 min and subsequently transferred into 70% ethanol. Bud scales were then carefully opened under a stereomicroscope, and the presence of localized brown spots indicating H_2_O_2_ accumulation was observed. Selected areas were photographed using a DP-20 digital camera system.

### 4.3. DAB Staining Scoring and Evaluation

The intensity of DAB staining in buds was evaluated using a three-grade semi-quantitative scoring system based on the intensity of brown coloration, which reflects H_2_O_2_ accumulation: 1 = weak, 2 = moderate, 3 = strong [46]. Scoring was performed by a single experienced observer using a stereomicroscope under constant lighting conditions, by directly examining each bud.

For each developmental stage, six buds from each chestnut cultivar were evaluated. The individual scores were summed and averaged to calculate the mean DAB score for each cultivar at each stage. These scores were then used to compare oxidative defense levels between cultivars. DAB scale values were statistically analyzed using one-way analysis of variance (ANOVA), and significant differences between groups were determined using Duncan’s multiple range test at a significance level of *p* < 0.05.

## 5. Conclusions

In this study, seasonal patterns of H_2_O_2_ accumulation and localization were monitored in dormant buds of chestnut cultivars with varying levels of resistance to the ACGW. Resistant cultivars showed a controlled and timely oxidative response, while susceptible cultivars maintained persistently high H_2_O_2_ levels without effective defense. Hybrid cultivars exhibited early defense activation, whereas pure *C. sativa* genotypes demonstrated a delayed but strong response. Additionally, the increase of H_2_O_2_ during bud break highlights its involvement in both defense signaling and developmental processes. These results emphasize the role of H_2_O_2_ as a key biochemical marker for understanding resistance mechanisms and provide a basis for future studies on temporal dynamics of plant defense.

## Figures and Tables

**Table 1:** DAB staining score in chestnut buds of chestnut cultivars.

Cultivars	Control	t1	t2	t3	t4	Sep	Oct	Dec	Jan	Feb	Mar	Apr
Bouche de Betizac	1.50 c	1.66 bc	2.16 ab	2.50 a	2.50 a	2.66 a	2.66 ^NS^	2.50 ^NS^	1.66 b	1.33 cd	2.66 ^NS^	1.16 ^NS^
Ertan	1.00 d	1.16 c	1.16 c	1.33 c	1.16 c	2.00 b	2.16	2.16	1.16 b	1.16 d	2.66	1.00
Tülü	1.66 bc	1.33 bc	1.83 b	1.66 bc	1.66 bc	2.50 ab	2.66	2.33	1.33 b	1.16 d	2.66	1.00
Maraval	2.50 a	1.66 bc	2.33 ab	2.50 a	2.50 a	2.66 a	2.50	2.33	2.33 a	1.83 bc	2.83	1.16
Marigoule	2.16 ab	2.33 a	2.50 a	2.33 a	2.50 a	2.66 a	2.50	2.50	2.50 a	2.66 a	3.00	1.33
Alimolla	2.16 ab	2.33 a	2.50 a	2.16 ab	2.33 a	2.33 ab	2.50	2.66	2.50 a	2.66 a	2.83	1.33
Sarıkestane	2.00 abc	1.83 ab	2.16 ab	2.00 ab	2.16 ab	2.33 ab	2.33	2.50	2.33 a	2.33 ab	2.83	1.00

Differences between means in each column were assessed using Duncan’s multiple range test at *p* < 0.05. Means followed by different lowercase letters are significantly different. NS: not significant.

## Data Availability

The original contributions presented in this study are included in the article. Further inquiries can be directed to the corresponding author.

## Abbreviations

The following abbreviations are used in this manuscript:

Am	Apical meristem
ACGW	Asian chestnut gall wasp
E	Eggs
G	Gall
Gf	Gall formation
HR	Hypersensitive response
H_2_O_2_	Hydrogen peroxide
DAB	3,3′-diaminobenzidine
ROS	Reactive oxygen species
R	Resistance
LS	Less susceptible
L	Larvae
Lv	Lateral vein
Lb	Leaf blades
Lp	Leaf promordia
Mv	Midvein
N	Necrosis
Np	Needle puncture
